# Development and Validation of the Food Consumption Rate Questionnaire (FCRQ): A Behaviorally Informed Measure of Eating-Rate Regulation

**DOI:** 10.3390/nu18162637

**Published:** 2026-08-12

**Authors:** Maria P. Koliou, Chrysoula Karaiskou, Charalampos Eleftheriadis, Dimitris Skalkos

**Affiliations:** 1 Laboratory of Food Chemistry, Department of Chemistry, University of Ioannina, 45110 Ioannina, Greece; m.koliou@uoi.gr; 2 Sidroco Holdings Ltd., Nicosia 1082, Cyprus; ckaraiskou@sidroco.com (C.K.); celeftheriadis@sidroco.com (C.E.)

**Keywords:** Food Consumption Rate Questionnaire (FCRQ), eating-rate regulation, behavioral nutrition, questionnaire development, questionnaire validation, factor analysis, confirmatory factor analysis, psychometric evaluation, interoceptive awareness, sensory engagement

## Abstract

**Background:** Eating-rate regulation is a multidimensional behavioral process shaped by oral-processing dynamics, interoceptive cues, sensory engagement, and contextual influences. Despite its relevance, no validated self-report instrument currently assesses these mechanisms. **Methods:** The Food Consumption Rate Questionnaire (FCRQ) was developed as a behaviorally informed tool for measuring eating-rate regulation. Item generation followed a theory-driven process supported by expert review, forward–backward translation, and pilot testing. Structural validity was examined through Exploratory Factor Analysis (Principal Axis Factoring, Promax rotation) and a theory-driven Confirmatory Factor Analysis evaluating the proposed four-domain model. Reliability was assessed using Cronbach’s α and corrected item–total correlations. Behavioral heterogeneity was explored using hierarchical and k-means cluster analysis. **Results:** A clear four-factor structure emerged—Slowness, Awareness of Eating Rate, External Factors, and Awareness & Enjoyment—explaining 42.77% of the variance. CFA indicated acceptable model fit (CFI = 0.93, TLI = 0.91, RMSEA = 0.052), supporting the adequacy of the theoretical structure. Reliability indices were consistent with the multidimensional nature of the construct. All four domains were positively correlated. Cluster analysis identified distinct behavioral profiles, illustrating the instrument’s discriminative capacity. **Conclusions:** The FCRQ provides the first behaviorally informed, psychometrically evaluated framework for assessing eating-rate regulation. The instrument demonstrates a coherent theoretical structure, satisfactory early-stage validity, and the ability to differentiate meaningful behavioral patterns. Continued refinement and validation across diverse populations will further establish the FCRQ as a robust measure for behavioral-nutrition research and practice.

## 1. Introduction

Eating rate, the speed at which individuals consume food, has increasingly been recognized as a fundamental behavioral determinant with broad implications for appetite regulation, energy intake, metabolic health, and long-term dietary patterns. Previous research has consistently shown that faster eating is associated with higher caloric intake, reduced oro-sensory exposure, impaired satiety signaling, and increased risk of overweight and obesity [1,2,3,4,5]. Rapid consumption has been linked to diminished interoceptive awareness and a shift toward automatic, externally driven eating, whereas slower eating enhances sensory exposure, increases enjoyment of food, and improves recognition of fullness [6,7,8]. Despite these well-established physiological and behavioral consequences, the underlying mechanisms shaping eating rate, particularly the interplay between internal awareness, emotional states, environmental cues, and social context, remain insufficiently understood.

This knowledge gap becomes particularly relevant when examining Generation Z, a cohort shaped by pervasive digital immersion, heightened emotional sensitivity, accelerated information processing, and rapidly shifting lifestyle patterns [9,10,11,12]. Young adults today frequently consume meals under conditions of distraction, time pressure, social facilitation, or environmental intensity, all of which have been shown to accelerate eating pace and disrupt satiety cues [13]. At the same time, Generation Z demonstrates increased interest in wellness, sustainability, and mindful eating, yet often struggles to translate these values into consistent behavioral regulation [14,15,16]. This paradox—between aspirational health-oriented attitudes and the realities of emotionally and environmentally driven eating—positions eating rate as a potentially critical behavioral marker for understanding contemporary youth.

Recent post-pandemic research has highlighted substantial variability in appetitive traits, emotional responsiveness, sensory tendencies, and regulatory capacities among young adults [13]. These findings have underscored the heterogeneity of *Generation Z university students in Greece, specifically those assessed at the University of Ioannina*, with distinct behavioral profiles emerging that reflect differences in emotional regulation, hedonic responsiveness, and openness to new foods. Moreover, behavioral emotional responses, such as emotional overeating and undereating, have been identified as key predictors of how young adults interpret hunger cues, suggesting that eating behavior in this cohort is shaped more by experiential and behavioral mechanisms than by cognitive insight [6].

Within this broader behavioral landscape—referring to the established variability in emotional regulation, sensory responsiveness, openness to new foods, and self-regulatory eating traits among young adults—the tempo of consumption emerges as a dimension that may integrate several of these tendencies [13]. Variability in eating pace observed across previous datasets, combined with the emotional and sensory patterns identified among young adults, suggests that eating rate may serve as a behavioral bridge linking emotional regulation, sensory responsiveness, and sustainable dietary choices [8,17,18]. The post-pandemic context—referring to the lifestyle disruptions, increased digital engagement, and altered social environments that emerged following COVID-19—further amplifies the relevance of this dimension, as these shifts have influenced not only what individuals eat but also how they eat. These converging trends point toward the need for a more targeted investigation of the behavioral and environmental factors influencing eating rate in Generation Z. Such an investigation requires tools capable of capturing both internal and external drivers of eating pace.

Recent empirical work from our group has contributed to a more integrated understanding of eating behavior in Generation Z by examining emotional, sensory, and regulatory determinants across large university samples [13]. Building on these findings, the development of the FCRQ represents a natural extension of this research trajectory, aiming to operationalize the behavioral dimension of eating rate with greater precision. Earlier studies mapped key appetitive traits and identified distinct emotional–sensory–regulatory profiles, while subsequent research further clarified the emotional mechanisms underlying hunger awareness [19]. Together, these findings underscore the multidimensional nature of eating behavior in contemporary youth and point to the need for instruments capable of capturing specific behavioral processes, including the tempo of consumption.

Although eating rate has been increasingly examined as a modifiable behavioral factor, existing research remains conceptually narrow. Many studies have focused primarily on physiological outcomes—such as energy intake, satiety responses, or metabolic markers—while giving comparatively less attention to the psychological, social, and environmental determinants that shape eating pace in real-life contexts. Social facilitation, for example, has been shown to increase food intake and accelerate eating speed in group settings [20]. Environmental intensity—such as noise, crowding, or digital distraction—can further disrupt interoceptive cues and promote rapid consumption [21]. Conversely, mindful eating practices, which emphasize awareness of sensory experience and internal cues, have been associated with slower eating, improved satiety regulation, and healthier food choices [22,23,24,25]. Yet, few studies have examined how these internal and external factors interact within the lived experiences of Generation Z, especially within Mediterranean cultural contexts where social eating norms, family traditions, and dietary patterns may exert unique influences.

This gap is particularly relevant in Mediterranean dietary cultures, where social eating norms, shared meals, and sensory-rich food traditions may uniquely interact with the pace of consumption—while still reflecting broader mechanisms observed among young adults globally.

The development of the FCRQ was therefore motivated by the absence of validated self-report measures assessing the behavioral tempo of eating. The theoretical foundation of the FCRQ draws upon established behavioral mechanisms known to influence eating rate, including oral processing dynamics, sensory exposure, interoceptive awareness, environmental intensity, and social facilitation. These mechanisms are supported by existing research on behavioral determinants of consumption tempo and are consistent with prior work examining emotional, sensory, and regulatory dimensions of eating behavior in Generation Z [2,8,26]. Accordingly, the formulation of the FCRQ items was behaviorally informed and grounded in an existing empirical and theoretical framework, rather than developed in isolation. Eating rate has been consistently linked to energy intake, oro-sensory exposure, satiety responsiveness, and long-term dietary patterns, yet existing tools do not capture the behavioral and environmental determinants that shape consumption pace. Given the multidimensional nature of eating rate—encompassing oral-processing dynamics, sensory engagement, interoceptive cues, and contextual influences—an instrument capable of assessing these mechanisms is required. The FCRQ was developed to provide a theoretically grounded, psychometrically structured assessment of eating-rate regulation.

Existing research highlights the behavioral complexity of eating rate, yet available tools do not capture the combined oral-processing, sensory, interoceptive, and contextual mechanisms that shape consumption tempo [27,28,29,30,31,32,33,34,35]. Instruments such as DEBQ, TFEQ, AEBQ, and mindful eating scales assess emotional, external, or cognitive–sensory traits but do not evaluate slowness in eating, interoceptive monitoring of pace, or environmental acceleration of consumption [29,30,31,36]. Given the multidimensional nature of eating rate and its links to energy intake, oro-sensory exposure, satiety responsiveness, and long-term dietary patterns [1,2,37] a validated self-report instrument capable of assessing both internal and external determinants of eating pace is needed. The FCRQ was developed to address this gap by providing a theoretically grounded, psychometrically structured assessment of the behavioral tempo of food consumption.

Although several validated instruments assess emotional eating, external eating, satiety responsiveness, mindful eating, or intuitive eating, these tools do not evaluate the behavioral tempo of food consumption [36,38,39,40,41]. As outlined in the instruments described above, existing questionnaires primarily assess psychological traits, attitudes, or general awareness rather than the specific behavioral mechanisms that shape eating speed. Constructs such as mindful eating or intuitive eating capture cognitive and sensory awareness but do not evaluate slowness, oral processing patterns, interoceptive monitoring of eating rate, or the extent to which environmental cues accelerate consumption. Likewise, measures of external eating and satiety responsiveness describe predispositions rather than real-time behavioral regulation. These limitations highlight the absence of tools that directly assess consumption tempo. Consequently, no available instrument captures the multidimensional behavioral processes underlying eating rate, including sensory exposure, contextual modulation, and moment-to-moment regulation of pace.

Recent literature increasingly conceptualizes eating rate as a complex behavioral construct shaped by oral processing dynamics, bite size, chewing tempo, sensory exposure, interoceptive cues, social facilitation, and environmental intensity [2,35,42,43,44]. Taken together, these mechanisms collectively shape the pace of consumption, yet they remain unmeasured by existing self-report tools. This multidimensionality underscores the need for an instrument capable of capturing both internal and external determinants of eating rates in real-life contexts. The FCRQ was therefore developed to address this gap by integrating behavioral, sensory, interoceptive, and contextual components into a single psychometrically structured framework.

To date, no validated instrument has systematically assessed the behavioral tempo of food consumption in Generation Z, particularly within real Mediterranean contexts.

Given the absence of tools capable of capturing these multidimensional influences on eating pace, a targeted assessment approach is required. Accordingly, the present study employs the newly developed Food Consumption Rate Questionnaire (FCRQ), an instrument designed to assess four domains central to the behavioral tempo of eating:

- Slowness in food consumption.
- Awareness of eating rate.
- External factors influencing pace.
- Awareness and enjoyment during eating.

Beyond its psychometric validation, the FCRQ also introduces a multidimensional conceptual framework of eating-rate regulation. This model integrates internal determinants (such as oral-processing dynamics, sensory engagement, and interoceptive monitoring) with external contextual influences (including environmental intensity and social facilitation), operationalizing these mechanisms into four distinct behavioral domains. By structuring eating-rate behavior within this integrated framework, the present study contributes a novel theoretical perspective that advances current understanding of how eating pace is shaped in real-life contexts, particularly among Generation Z.

As described in its development, the FCRQ was designed to advance understanding in an underexplored area of eating behavior and to fill a gap in the international literature regarding behavioral eating speed. Applying the FCRQ to a large sample of Generation Z students at the University of Ioannina allows the present study to extend emerging evidence on the emotional, sensory, and regulatory dimensions of eating behavior in young adults [45].

Within this evolving framework, the present study advances the field by focusing specifically on the behavioral tempo of consumption. A conceptual model illustrating the interplay between emotional, sensory, regulatory, and contextual factors influencing eating rate is provided in Supplementary Figure S1. This study represents the continuation and deepening of an evolving scientific narrative. The progression of this research program can be conceptualized as follows:

1. Mapping appetitive traits in young adults.
2. Identifying behavioral heterogeneity and distinct emotional–sensory–regulatory profiles.
3. Modeling emotional mechanisms that shape hunger, satiety, and eating tendencies.
4. Examining the behavioral tempo of consumption as a potentially foundational dimension of eating behavior in contemporary youth.

By quantifying eating-rate behaviors and exploring their internal and external determinants, the present study aims to enrich the emerging multidimensional framework of sustainable eating behavior among young adults. Understanding how Generation Z perceives, regulates, and responds to contextual cues during meals is essential for informing targeted interventions that foster healthier, more mindful, and more sustainable dietary practices.

## 2. Materials and Methods

### 2.1. Study Design and Participants

This cross-sectional study was conducted at the University of Ioannina, a large public institution in northwestern Greece. The target population consisted of actively enrolled undergraduate students belonging to Generation Z (born 1995–2010). As documented in previous studies of the research group, inactive enrollment is common in Greek higher education; therefore, the effective active student population was estimated at approximately 18,000 individuals, of whom roughly 14,500 fell within the Generation Z age range.

The present investigation forms part of a broader research program examining emotional, sensory, and behavioral determinants of eating behavior among young adults, as reflected in recent empirical work on Generation Z eating profiles [13]. Data collection for the current study took place in March 2026, with voluntary and anonymous participation.

The final sample consisted of 555 participants. All questionnaires were screened using predefined exclusion criteria, and only valid, fully completed responses were retained for analysis. Inclusion criteria were:

- Active enrollment at the University of Ioannina.
- Age consistent with Generation Z.
- Full completion of the questionnaire.

The demographic section included an item on age, which participants could skip if they wished. The study invitation email clearly specified the eligible age range corresponding to Generation Z, minimizing the likelihood of responses from individuals outside this group. During data screening, only questionnaires from respondents whose reported age fell within the predefined eligibility criteria were retained for analysis. Participants self-reported the absence of medically diagnosed eating disorders, as the study focused on non-clinical behavioral patterns.

A conceptual model illustrating the interplay between emotional, sensory, regulatory, and contextual factors influencing eating rate is provided in Supplementary Figure S1.

### 2.2. Instrument: Food Consumption Rate Questionnaire (FCRQ)

The Food Consumption Rate Questionnaire (FCRQ) is a 20-item instrument developed within the framework of the doctoral dissertation to assess behavioral and environmental determinants of eating rate. Items are rated on a five-point Likert scale (1 = Strongly disagree, 5 = Strongly agree), with respondents evaluating each statement “compared to usual.” The full item wording of the FCRQ is provided in Supplementary Table S1.

The FCRQ comprises four theoretically grounded domains:

- **Slowness in Food Consumption (5 items)—F1:** perceived eating pace and comparative slowness (includes one reverse-coded item). Slower eating has been associated with increased oro-sensory exposure and improved satiety signaling [1,46].
- **Awareness of Eating Rate (5 items)—F2:** interoceptive and cognitive awareness of changes in eating speed. Awareness of internal cues is central to mindful eating and self-regulation [47,48].
- **External Factors and Eating Rate (5 items)—F3:** influence of social presence, environmental intensity, and time pressure. Social facilitation and environmental distraction are known to accelerate eating [35,49].
- **Awareness and Enjoyment of Eating (5 items)—F4:** mindful engagement, sensory enjoyment, and recognition of fullness, which have been linked to slower consumption and enhanced satiety responsiveness [34,50].

The FCRQ was administered in Greek, which was the primary language of data collection. For manuscript presentation and international dissemination, the instrument was also translated into English using a forward–backward procedure to ensure linguistic and conceptual equivalence between versions [51,52]. Content validity was evaluated by experts in food chemistry, behavioral nutrition, and psychometrics. The complete questionnaire is provided in the Supplementary Materials.

The development of the FCRQ was also informed by previous empirical work conducted by our research group, which systematically examined emotional, sensory, and regulatory determinants of eating behavior among young adults [13,19]. These studies identified distinct behavioral profiles, variability in interoceptive awareness, and the influence of contextual and environmental cues on eating tendencies, highlighting the multidimensional nature of eating behavior in Generation Z. The emerging patterns from this body of work provided the conceptual foundation for defining the four domains of the FCRQ and guided the theory-driven formulation of the 20 items. In this context, the present instrument represents a natural extension of the research group’s published findings, offering a more detailed examination of a specific behavioral dimension that previously showed scientific relevance.

The initial development of the FCRQ did not rely on a large item pool or exploration factor analyses typically used in broad psychometric scale construction. Instead, the 20 items were generated through a theory-driven process grounded in four established behavioral frameworks: oral processing dynamics, interoceptive awareness, environmental modulation of eating, and mindful sensory engagement. Item wording was informed by (a) targeted literature review, (b) empirical patterns identified in previous studies of the research group, and (c) internal conceptual appraisal focusing on clarity, specificity, and theoretical alignment. Following this stage, content experts reviewed the preliminary item set to ensure conceptual relevance and domain coverage, and minor refinements were applied accordingly. Additional details regarding item appraisal and refinement are provided in Supplementary Tables S2 and S3.

In the present study, the instrument demonstrated adequate internal consistency and supportive evidence for the proposed factor structure based on Confirmatory Factor Analysis (CFA), as detailed in Section 2.7, Section 2.8 and Section 2.9 and the Supplementary Materials.

#### Rationale for Instrument Development

The FCRQ was developed to operationalize four behavioral domains identified as central to the tempo of eating: slowness in consumption, awareness of eating rate, external modulation of pace, and mindful sensory engagement. The instrument’s structure reflects a theory-driven approach focused on capturing specific behavioral mechanisms—oral processing, sensory engagement, interoceptive cues, and contextual influences—rather than broad psychological traits. This methodological rationale guided item formulation and ensured alignment with the underlying behavioral constructs.

### 2.3. Procedure

Data was collected electronically using the Google Forms platform. The questionnaire required less than 10 min to complete. No incentives were offered.

### 2.4. Data Handling

Data cleaning and preparation followed standardized procedures applied in previous studies of the research group to ensure methodological consistency.

1. Missing data: Cases with substantial missingness were excluded using listwise deletion. A very small number of isolated item-level omissions were identified across otherwise complete questionnaires; these omissions were minimal, non-systematic [53,54], and did not affect the analytical workflow or the validity of subsequent analyses. Sensitivity checks confirmed that inclusion versus exclusion of these isolated omissions did not materially alter the results.
2. Reverse-coded item: One item in the Slowness in Food Consumption domain (“I often finish my meals very quickly”) was reverse-coded prior to analysis to ensure consistent scoring direction across all items.
3. Variable preparation: Likert-type responses were treated as approximately interval-level data due to the large sample size [55].
4. Factor scores: Scores for each FCRQ domain were computed as the means of the corresponding items. In addition to domain scores, an overall FCRQ score was computed as the mean of all 20 items, where appropriate.

All data were processed using IBM SPSS Statistics (Version 25.0).

### 2.5. Ethical Considerations

The study adhered to the ethical principles of the Declaration of Helsinki and complied with GDPR. Participation was voluntary, anonymous, and based on informed consent, with no collection of personally identifiable information. Ethical authorization for conducting the study was granted by the Bureau of Personal Data Protection of the University of Ioannina (Approval No. 5226/12-02-2026), issued on 12 February 2026, under the institutional procedure for non-commercial, non-clinical behavioral research.

### 2.6. Statistical Analysis

Descriptive statistics (means, standard deviations) were calculated for all items. Sampling adequacy for factor analysis was assessed using the Kaiser–Meyer–Olkin (KMO) statistic [56] and Bartlett’s test of sphericity [57].

An Exploratory Factor Analysis (EFA) was conducted using Principal Axis Factoring with Promax rotation, an oblique rotation method appropriate for correlated behavioral constructs [58,59]. Factors with eigenvalues > 1.0 were retained, and items with loadings ≥ 0.50 were included in the final solution.

A Confirmatory Factor Analysis (CFA) was conducted to evaluate the hypothesized four-factor structure of the Food Consumption Rate Questionnaire (FCRQ). The model was specified according to the theoretically proposed domains and estimated using maximum likelihood procedures.

The CFA demonstrated an acceptable fit to the data (Table 1), with χ^2^/*df* = 2.51, CFI = 0.93, TLI = 0.91, RMSEA = 0.052 (90% CI: 0.046–0.059), and SRMR = 0.061. Standardized factor loadings ranged from 0.37 to 0.91 across the four latent constructs, indicating adequate item representation of each factor.

Convergent validity was assessed using the Average Variance Extracted (AVE) and Composite Reliability (CR). AVE values ranged from 0.27 to 0.67, while CR values ranged from 0.62 to 0.91. The Slowness and Awareness & Enjoyment factors demonstrated satisfactory convergent validity (AVE ≥ 0.45; CR ≥ 0.80), whereas Awareness and External Factors showed lower AVE values, which are nevertheless acceptable for behavioral constructs.

Discriminant validity was supported through the Heterotrait–Monotrait ratio (HTMT) and the Fornell–Larcker criterion. All HTMT values were below 0.85, indicating clear separation between constructs. Additionally, the square root of AVE for each factor exceeded its inter-factor correlations, further confirming discriminant validity.

Overall, the CFA results support the four-factor theoretical structure of the FCRQ and confirm the adequacy of its psychometric properties. Detailed CFA outputs—including standardized factor loadings, AVE/CR values, HTMT ratios, and Fornell–Larcker matrices—are provided in the Supplementary Materials (Tables S4–S7) [59,60].

Cluster analysis (hierarchical followed by k-means) was used to explore potential behavioral subgroups based on the four FCRQ domains. All analyses were performed using IBM SPSS Statistics (Version 25.0).

### 2.7. Reliability Analysis

Internal consistency reliability was assessed using Cronbach’s alpha, with values ≥ 0.70 considered acceptable [61,62]. Item–total correlations, corrected item–total correlations, and ‘alpha if item deleted’ statistics were examined to evaluate the contribution of each item to its respective scale, following established psychometric guidelines [63]. Reliability analyses were conducted separately for each FCRQ domain, consistent with the theoretical structure of the instrument.

### 2.8. Exploratory Factor Analysis (Supplementary Material)

Detailed EFA outputs—including factor loadings, communalities, eigenvalues, scree plot, and full KMO/Bartlett statistics—are provided in the Supplementary Materials to ensure transparency and reproducibility.

### 2.9. Convergent and Discriminant Validity

Convergent validity was assessed using Average Variance Extracted (AVE) and Composite Reliability (CR), while discriminant validity was evaluated using the Fornell–Larcker criterion and the Heterotrait–Monotrait (HTMT) ratio. All indices were computed using standardized factor loadings from the CFA model, which was used to evaluate the proposed theoretical structure of the instrument. Detailed results are presented in Supplementary Tables S8 and S9.

## 3. Results

### 3.1. Sample Sociodemographic Profile

The final analytical sample consisted of N = 555 young adults. Most participants were female (76.2%), while males accounted for 23.4% of the sample. The majority were 19–22 years old (67.6%), followed by those aged 23–28 years (22.9%). Most respondents were full-time students (77.8%), whereas 22.0% combined studies with part-time employment. Regarding geographical distribution, participants primarily originated from Western Greece (33.5%), Northern Greece (26.3%), and Central Greece including Athens (25.8%). A detailed breakdown of all sociodemographic variables is provided in Supplementary Table S10, ensuring full transparency of sample characteristics.

### 3.2. Descriptive Statistics of the Four FCRQ Dimensions

Descriptive statistics for the four FCRQ dimensions are presented in Table 2. Mean scores ranged from 2.84 (Slowness) to 3.55 (Enjoyment), indicating moderate to moderately high levels across constructs. Standard deviations (0.57–0.74) reflected adequate variability within the sample. Skewness and kurtosis values for all dimensions fell within acceptable ranges (±2), supporting the assumption of approximate normality. Item-level descriptive statistics for all FCRQ items are provided in Supplementary Table S11.

Gender-stratified item-level descriptive statistics for all 20 FCRQ items are presented in Supplementary Table S12, illustrating response patterns across male and female participants.

### 3.3. Factor Structure of the FCRQ

Exploratory factor analysis using Principal Axis Factoring with Promax rotation supported a four-factor solution consistent with the conceptual structure of the FCRQ. The four retained factors had eigenvalues ≥1 and collectively explained 53.38% of the total variance based on initial eigenvalues, while 43.14% of the variance was retained after extraction (Table 3).

The factor solution aligned with the predefined dimensions of the instrument. The pattern matrix indicated a clear and interpretable structure, with all items loading strongly on their expected factors (Supplementary Table S13).

- **Factor 1–Slowness in Food Consumption**: Q1_1–Q1_5 (loadings 0.469–0.914).
- **Factor 2–Awareness of Eating Rate**: Q2_1–Q2_5 (loadings 0.372–0.693).
- **Factor 3–External Factors Influencing Eating Rate**: Q3_1–Q3_5 (loadings 0.314–0.667).
- **Factor 4–Awareness and Enjoyment of Eating**: Q4_1–Q4_5 (loadings 0.465–0.827).

Overall, the extracted factors represent related yet distinct dimensions of food-consumption rate behaviors and perceptions.

Inter-factor correlations derived from the Promax-rotated solution ranged from −0.034 to 0.297, indicating low to moderate associations among the latent constructs (Table 4). This correlation pattern supports the use of an oblique rotation and confirms that the extracted factors represent related yet distinct dimensions of the FCRQ.

Beyond the exploratory phase, the structural validity of the four-factor model was further examined through Confirmatory Factor Analysis (CFA). CFA results supported the adequacy of the proposed theoretical structure, demonstrating acceptable model fit and confirming that each factor captured a coherent behavioral dimension of food consumption rate.

Taken together, the CFA findings provide strong psychometric support for the four-factor structure of the FCRQ, with each dimension reflecting a theoretically meaningful and empirically distinguishable construct: Slowness in Food Consumption, Awareness of Eating Rate, External Factors & Eating Rate, and Awareness & Enjoyment of Eating.

### 3.4. Convergent and Discriminant Validity

Convergent validity was supported for *Slowness in Food Consumption* (AVE = 0.57, CR = 0.89) and *Awareness and Enjoyment of Eating* (AVE = 0.49, CR = 0.82). Convergent validity indices for *Awareness of Eating Rate* (AVE = 0.41, CR = 0.74) and *External Factors and Eating Rate* (AVE = 0.29, CR = 0.63) were lower but acceptable given the behavioral specificity of these constructs. Discriminant validity was fully supported: √AVE values exceeded all inter-factor correlations, and HTMT ratios ranged from 0.17 to 0.34, well below the recommended threshold of 0.85. Full validity indices are presented in Supplementary Tables S8 and S9.

### 3.5. Reliability Analysis

Internal consistency reliability was assessed using Cronbach’s α and corrected item–total correlations (CITC) for each FCRQ factor and for the total scale (Table 5). Reliability estimates for the four factors ranged from α = 0.777 to α = 0.796, indicating satisfactory internal consistency across dimensions. CITC values were positive and within acceptable ranges, supporting item coherence within each factor. Cronbach’s α if item deleted values showed minimal improvement across items, confirming the stability of the factor structures.

The total 20-item scale demonstrated good reliability (α = 0.796), with CITC values ranging from 0.070 to 0.585. These results support the multidimensional structure of the instrument and indicate that the FCRQ captures related yet distinct behavioral domains.

Modest variability in CITC values—particularly within Slowness in Food Consumption, Awareness of Eating Rate, and External Factors & Eating Rate—is expected in early-stage behavioral instrument development. These domains reflect heterogeneous behavioral tendencies rather than narrow psychological traits, which naturally results in lower shared variance among items. In such contexts, structural validity is considered the primary indicator of adequacy.

The present study employed both exploratory and confirmatory approaches. The EFA provided an initial examination of item clustering and suggested a four-factor configuration consistent with the theoretically proposed domains. Subsequently, the CFA evaluated the hypothesized structure and demonstrated acceptable model fit, supporting the conceptual integrity and structural coherence of the instrument.

Item-level diagnostics (CITC, α if item deleted) were reported to ensure transparency and to guide future refinement. Although removing individual items would increase internal consistency, doing so would narrow the conceptual breadth of the domains. Given the multidimensional nature of eating-rate behaviors, theoretical coverage and structural coherence were prioritized, with future research planned to refine item wording and evaluate alternative item combinations in independent samples.

### 3.6. Correlations Among the Four FCRQ Dimensions

Pearson correlation coefficients were calculated to examine associations among the four FCRQ dimensions (Table 6). All correlations were positive and statistically significant (*p* < 0.01), indicating coherent relationships among the underlying constructs. Correlation coefficients ranged from r = 0.146 to r = 0.306, reflecting small to moderate associations.

The strongest association was observed between External Factors & Eating Rate and Awareness & Enjoyment of Eating (r = 0.306), whereas the weakest was between Slowness in Food Consumption and External Factors & Eating Rate (r = 0.146). These patterns align with the theoretical structure of the instrument, supporting both convergent and discriminant validity and confirming that the four dimensions capture related yet distinct aspects of eating-rate behavior.

When considered alongside the correlation matrix, the CFA findings provide additional evidence for the structural coherence of the FCRQ and reinforce the multidimensional nature of the four behavioral domains.

### 3.7. Cluster Analysis

To examine heterogeneity in eating-rate behavior, a two-step clustering procedure was applied using the four FCRQ domains: Slowness, Awareness of Eating Rate, External Factors, and Awareness & Enjoyment. Hierarchical clustering (Ward’s method) initially indicated a four-cluster solution, which was subsequently refined using k-means clustering. The final cluster centers are presented in Table 7.

To examine heterogeneity in eating-rate behavior, a two-step cluster analysis was conducted using the four FCRQ domains: Slowness, Awareness, External Factors, and Enjoyment. Hierarchical clustering (Ward’s method) initially suggested a four-cluster solution, which was subsequently refined using k-means clustering. The final cluster centers are presented in Table 7.

Cluster analysis was conducted to examine heterogeneity in eating-rate behavior using the four FCRQ domains: Slowness in Food Consumption, Awareness of Eating Rate, External Factors & Eating Rate, and Awareness & Enjoyment of Eating. Hierarchical clustering (Ward’s method) initially indicated a four-cluster solution, which was subsequently refined using k-means clustering. The final cluster centers are presented in Table 6.

**Cluster 1 (n = 170)** was characterized by relatively high scores across all four domains, reflecting slower eating, elevated awareness, stronger external modulation, and higher enjoyment during eating.

**Cluster 2 (n = 111)** showed moderate slowness and external influence, lower awareness, and high enjoyment, representing a mixed behavioral profile with reduced self-monitoring but preserved positive affect during eating.

**Cluster 3 (n = 85)** exhibited lower slowness, lower external influence, and reduced enjoyment, combined with moderate awareness, indicating a faster and less positively experienced eating pattern.

**Cluster 4 (n = 188)** demonstrated high awareness, moderate slowness, and elevated external influence, together with moderate enjoyment, suggesting a profile characterized by strong self-monitoring and sensitivity to environmental cues.

The four-cluster solution demonstrated conceptual interpretability and statistical stability. The clusters captured meaningful variability in eating-rate behaviors, illustrating how the four FCRQ domains combine into distinct behavioral profiles within the student population. When considered alongside the CFA findings, reliability indices, and inter-dimension correlations, the cluster results further support the multidimensional nature of the FCRQ.

The cluster analysis should be interpreted as an exploratory application rather than a validation procedure. Its purpose was to illustrate potential behavioral patterns derived from the four FCRQ domains. Because external correlates were not included, the clusters cannot yet be evaluated in terms of behavioral, nutritional, or anthropometric relevance. Future research will incorporate external variables—such as dietary behaviors, anthropometric indicators, and objective measures of eating rate—to assess the practical and predictive utility of the FCRQ and to determine whether these clusters represent meaningful behavioral subgroups.

## 4. Discussion

### 4.1. Summary of Key Findings

The present study examined the behavioral tempo of food consumption among Generation Z university students using the newly developed Food Consumption Rate Questionnaire (FCRQ). Four key findings emerged. First, the FCRQ demonstrated a clear and theoretically coherent four-factor structure, as supported by the confirmatory factor analysis, capturing **Slowness in Food Consumption**, **Awareness of Eating Rate**, **External Factors & Eating Rate**, and **Awareness & Enjoyment of Eating**. Second, reliability indices varied across domains, reflecting the heterogeneous nature of early-stage behavioral constructs. Third, the four dimensions were positively correlated, indicating interconnected yet distinct mechanisms underlying eating-rate regulation. Finally, cluster analysis identified four meaningful behavioral profiles, illustrating heterogeneity in how young adults regulate their eating pace and respond to internal and external cues.

Collectively, these findings support the multidimensional nature of eating-rate behavior and highlight the potential of the FCRQ to capture nuanced behavioral patterns in contemporary youth. However, the instrument remains in an early developmental stage, and further validation is required before definitive conclusions can be drawn.

### 4.2. Comparison with Previous Literature

The patterns observed across the four FCRQ dimensions are consistent with the theoretical structure of the instrument, supporting its ability to capture interconnected behavioral mechanisms underlying eating rate. The positive associations among slowness, sensory engagement, interoceptive awareness, and contextual sensitivity align with the conceptual model on which the FCRQ was developed, indicating that these domains operate synergistically in real-life eating contexts.

The behavioral clusters identified in the present study further reinforce the instrument’s discriminative capacity. Profiles characterized by externally driven pace or reduced enjoyment correspond to patterns previously described in emotional–sensory–regulatory research, whereas clusters reflecting slow, mindful engagement demonstrate the FCRQ’s sensitivity to regulatory and sensory-based eating tendencies. Overall, the findings suggest that the FCRQ effectively differentiates meaningful behavioral profiles and captures the multidimensional nature of eating rate regulation.

### 4.3. Theoretical Implications

The findings contribute to a growing theoretical framework positioning eating rate as a behavioral bridge linking emotional regulation, sensory responsiveness, and environmental influences. The multidimensional structure of the FCRQ supports the conceptualization of eating rate as a constellation of interrelated processes shaped by internal awareness, hedonic engagement, and contextual cues.

The heterogeneity observed in the cluster analysis reinforces the idea that eating rate is embedded within broader behavioral ecosystems. For some individuals, eating pace appears driven primarily by interoceptive and sensory mechanisms, whereas for others it is shaped more strongly by environmental or emotional factors. This complexity aligns with post-pandemic behavioral research showing that young adults navigate eating environments characterized by heightened digital immersion, fluctuating emotional states, and shifting social norms.

Although the total variance explained after factor extraction was modest, this level is typical for early-stage behavioral instruments with multidimensional constructs, where theoretical coherence and clean loading patterns are considered stronger indicators of structural validity than absolute variance explained.

The biological and behavioral significance of eating-rate regulation further contextualizes these findings. Faster eating reduces oro-sensory exposure and shortens the duration of oral processing, limiting the activation of cephalic-phase satiety signals and delaying the onset of fullness. This pattern is consistent with evidence showing that rapid consumption disrupts interoceptive monitoring, promotes externally driven eating, and increases energy intake. Conversely, slower eating enhances sensory exposure, prolongs oro-sensory stimulation, and facilitates more accurate detection of satiety cues, supporting regulatory mechanisms that promote healthier intake patterns. The FCRQ domains reflect these mechanisms: slowness corresponds to extended oral processing, awareness of eating rate aligns with interoceptive monitoring, enjoyment relates to hedonic sensory engagement, and external factors capture contextual accelerators of consumption. Thus, the multidimensional structure observed in the present study mirrors established biological pathways underlying satiety regulation and eating-rate control.

Beyond the four distinct clusters, an interpretive synthesis suggests that these profiles may be grouped into two broader behavioral sub-types:

(A) **Mindful–Positive Regulation (Clusters 1 and 2):** characterized by slower or moderate eating pace, higher enjoyment, and varying degrees of awareness. These profiles reflect more regulated and positively experienced eating behaviors.
(B) **Fast–Context-Driven Regulation (Clusters 3 and 4):** marked by faster eating, lower enjoyment, and stronger sensitivity to contextual or environmental cues. These profiles represent more rapid, externally modulated eating tendencies.

These two higher-order sub-groups provide a preliminary conceptual framework for understanding how combinations of slowness, awareness, enjoyment, and external modulation coalesce into broader eating-rate tendencies among young adults. Although exploratory, this synthesis may guide future validation studies and support the development of targeted behavioral interventions.

From a behavioral perspective, these profiles may reflect distinct regulatory pathways with meaningful implications for dietary patterns and long-term health. Individuals in the Mindful–Positive Regulation subgroup typically exhibit slower eating, greater sensory engagement, and more accurate interoceptive monitoring—mechanisms associated with improved satiety responsiveness, lower energy intake, and healthier dietary choices. In contrast, the Fast–Context Driven Regulation subgroup demonstrates patterns linked to rapid consumption, reduced sensory exposure, and heightened reactivity to environmental cues, all of which have been associated with overeating, dysregulated intake, and increased obesity risk in the previous literature. These behavioral profiles therefore offer a useful framework for designing targeted interventions: mindful–positive eaters may benefit from reinforcement strategies that sustain existing regulatory behaviors, whereas fast–context-driven eaters may require interventions focused on sensory enhancement, interoceptive training, and environmental restructuring. Integrating these profiles into behavioral nutrition programs could support more personalized and effective approaches to improving eating-rate regulation among young adults.

#### Conceptual Relationship Between FCRQ Dimensions and Eating Rate

Although the four FCRQ dimensions were theoretically derived from mechanisms known to influence eating pace, the present study did not include objective behavioral measures of eating rate, which will be incorporated in subsequent validation phases to strengthen criterion and convergent validity (e.g., bite frequency, chewing tempo, meal duration). As a result, it remains uncertain whether the FCRQ captures determinants of eating rate specifically or broader eating-related tendencies. Future validation studies incorporating objective behavioral indicators are needed to clarify the conceptual boundaries of the instrument.

Beyond the empirical findings, the present study introduces a multidimensional conceptual model of eating-rate regulation. This model integrates internal determinants (oral-processing dynamics, sensory engagement, interoceptive monitoring) with external contextual influences (environmental intensity, social facilitation) and organizes them into four distinct behavioral domains. By articulating eating rate within this integrated framework, the study advances current theoretical understanding of how pace of consumption emerges from the interaction of internal and external mechanisms.

### 4.4. Practical Implications

The identification of distinct eating-rate profiles has important implications for intervention design. Mindful slow-enjoyment eaters may benefit from reinforcement strategies that sustain existing healthy behaviors, whereas fast low-enjoyment eaters may require targeted interventions focusing on sensory engagement, interoceptive training, and reduction in environmental distractions. Externally driven high-awareness eaters may respond well to environmental restructuring strategies, such as reducing digital stimuli during meals or modifying social eating contexts.

At a broader level, the FCRQ can support campus-based nutrition programs by helping practitioners identify students who may be at risk for dysregulated eating patterns. Integrating eating-rate awareness into nutrition education may promote healthier, more mindful, and more sustainable dietary practices among young adults.

### 4.5. Strengths and Limitations

A major strength of this study is the large sample size and the use of a newly developed instrument designed specifically to capture the behavioral tempo of eating. The combination of factor analysis, reliability assessment, and cluster modeling provides a comprehensive evaluation of the FCRQ’s psychometric and behavioral utility.

The modest internal consistency observed in certain domains should be interpreted within the context of early-stage behavioral instrument development. Slowness in eating represents a heterogeneous behavioral construct encompassing multiple micro-behaviors (e.g., bite size, chewing tempo, pauses), which do not necessarily co-occur consistently across individuals. Psychometric literature emphasizes that in early phases of behavioral scale development, structural validity and conceptual coverage take precedence over internal consistency, especially when the construct is inherently multidimensional.

Although removing item Q1_3 would increase Cronbach’s α, the item reflects a theoretically central behavioral expression and demonstrated adequate structural validity. Consistent with psychometric recommendations, item deletion was avoided to preserve conceptual breadth.

Several limitations should be acknowledged. The cross-sectional design precludes temporal stability assessment, and self-reported measures may be subject to recall or social desirability bias. The sample consisted of university students from a single institution, limiting generalizability. Gender imbalance (76.2% women) may have influenced cluster prominence, and future studies should examine whether similar profiles emerge in more gender-balanced samples. The cluster analysis was exploratory and not externally validated, and future research should employ confirmatory approaches such as latent profile analysis or split-sample replication.

Finally, the study did not include objective measures of eating rate or external validation indicators, limiting the ability to assess convergent, discriminant, and criterion validity. These forms of validity will be addressed in subsequent phases of instrument development.

Taken together, these findings position the FCRQ not only as an emerging psychometric tool but also as a theoretically grounded framework that conceptualizes eating-rate behavior across four interrelated domains. This dual contribution—conceptual and psychometric—represents a key advancement in the study of eating-rate regulation among young adults.

### 4.6. Future Research Directions

Future research should examine the stability of the identified clusters over time and explore how eating-rate profiles relate to dietary quality, emotional regulation, and metabolic outcomes. Ecological momentary assessment could provide real-time insights into how environmental cues and emotional states influence eating pace in naturalistic settings. Cross-cultural validation of the FCRQ would clarify the role of cultural norms and social eating practices in shaping eating-rate behaviors. Finally, intervention studies are needed to determine whether modifying eating rates can meaningfully improve satiety responsiveness, dietary choices, and long-term health outcomes among young adults.

Overall, the present study provides an initial foundation for the development of the FCRQ, but additional psychometric work is required before the instrument can be considered fully validated.

## 5. Conclusions

The present study offers initial evidence supporting the development and early validation of the Food Consumption Rate Questionnaire (FCRQ). The instrument demonstrated a clear and theoretically coherent four-factor structure—Slowness, Awareness of Eating Rate, External Factors, and Awareness & Enjoyment—consistent with its conceptual foundations. Confirmatory factor analysis supported the adequacy of this structure, and the internal associations among the four domains indicate that the FCRQ captures interconnected behavioral mechanisms underlying eating rate regulation.

The identification of distinct behavioral profiles further illustrates the instrument’s discriminative capacity, suggesting that the FCRQ can meaningfully differentiate patterns of eating-rate regulation in real-life contexts. These findings highlight the potential utility of the FCRQ as a psychometrically structured tool for assessing the behavioral tempo of food consumption.

Future research should continue the psychometric refinement of the instrument, examine its stability across diverse populations, and explore its applicability in intervention and behavioral-nutrition settings. Overall, the FCRQ provides a foundational step toward establishing a validated measure of eating-rate regulation suitable for research and practice.

Beyond its initial validation, the present study also introduces a multidimensional conceptual model of eating-rate regulation. By integrating internal determinants (such as oral-processing dynamics, sensory engagement, and interoceptive monitoring) with external contextual influences (including environmental intensity and social facilitation), the FCRQ operationalizes these mechanisms into four distinct behavioral domains. This conceptual contribution provides a structured theoretical framework for understanding how eating-rate behaviors emerge and interact in real-life contexts, particularly among Generation Z.

Taken together, these findings position the FCRQ as a promising instrument for assessing the behavioral regulation of eating rate. However, its broader application requires further validation across diverse populations and against objective indicators of eating rate (e.g., bite frequency, chewing tempo, meal duration). Continued refinement and cross-contextual testing will be essential before the FCRQ can be widely implemented in behavioral nutrition research and practice.

## Figures and Tables

**Table 1:** CFA Model Fit Indices for the Four-Factor FCRQ Model.

Fit Index	Value	Criterion
χ^2^/*df*	2.51	<3 acceptable
CFI	0.93	≥0.90
TLI	0.91	≥0.90
RMSEA	0.052 (90% CI: 0.046–0.059)	≤0.08
SRMR	0.061	≤0.08

**Note.** Model fit criteria based on established guidelines.

**Table 2:** Descriptive statistics for the four FCRQ dimensions. [Values reflect agreement on a 5-point Likert scale (1 = strongly disagree, 5 = strongly agree)].

Dimension	Mean	SD	Min	Max	Skewness	Kurtosis
Slowness in Food Consumption	2.84	0.59	1.60	4.20	0.30	−0.75
Awareness of Eating Rate	3.52	0.58	1.20	5.00	−0.65	0.97
External Factors and Eating Rate	3.29	0.57	1.00	5.00	−0.23	0.58
Awareness and Enjoyment of Eating	3.55	0.74	1.00	5.00	−0.26	−0.14

**Table 3:** Total Variance Explained (Principal Axis Factoring). [Only factors with eigenvalues ≥1 were retained].

Factor	Initial Eigenvalue	% Variance	Cumulative %	Extraction SS Loadings	% Variance	Cumulative %
Factor 1	4.602	23.01	23.01	4.238	21.19	21.19
Factor 2	2.629	13.15	36.16	2.114	10.57	31.76
Factor 3	1.990	9.95	46.11	1.425	7.12	38.88
Factor 4	1.454	7.27	53.38	0.852	4.26	43.14

**Table 4:** Factor Correlation Matrix (Promax Rotation).

Factor	1	2	3	4
Factor 1	1.000	0.297	0.191	−0.034
Factor 2	0.297	1.000	0.159	0.276
Factor 3	0.191	0.159	1.000	0.078
Factor 4	−0.034	0.276	0.078	1.000

**Table 5:** Reliability indices (based on Item–Total Statistics).

Factor	Cronbach’s α	CITC (Min–Max)	α if Item Deleted (Range)	N Items
Factor 1	0.777	0.375–0.579	0.759–0.775	5
Factor 2	0.788	0.151–0.295	0.780–0.788	5
Factor 3	0.796	−0.028–0.488	0.767–0.796	5
Factor 4	0.784	0.237–0.585	0.761–0.784	5
Total scale	0.796	0.070–0.585	0.759–0.796	20

**Table 6:** Pearson correlations among the four FCRQ dimensions.

Dimension	Slowness	Awareness	External	Enjoyment
Slowness in Food Consumption	1	0.153 **	0.146 **	0.239 **
Awareness of Eating Rate	0.153 **	1	0.167 **	0.162 **
External Factors & Eating Rate	0.146 **	0.167 **	1	0.306 **
Awareness & Enjoyment of Eating	0.239 **	0.162 **	0.306 **	1

** All correlations were positive and statistically significant (*p* < 0.01).

**Table 7:** Final Cluster Centers for the Four FCRQ Domains.

Domain	Cluster 1 (n = 170)	Cluster 2 (n = 111)	Cluster 3 (n = 85)	Cluster 4 (n = 188)
Slowness in Food Consumption	3.28	2.61	2.41	2.76
Awareness of Eating Rate	3.82	2.93	3.08	3.78
External Factors & Eating Rate	3.50	3.33	2.73	3.33
Awareness & Enjoyment of Eating	4.17	3.94	2.55	3.21

**Note**. Values represent final cluster centers (mean scores) derived from k-means clustering using the four FCRQ domains.

## Data Availability

The data presented in this study is available on request from the corresponding author due to legal reasons.

## Supplementary Materials

The following supporting information can be downloaded at: https://www.mdpi.com/article/10.3390/nu18162637/s1, Figure S1: Conceptual model illustrates the emotional, sensory, regulatory, and contextual factors influencing eating rate, along with their downstream effects on behavioral outcomes, dietary choices, weight status, and overall health impacts; Table S1: Questionnaire on Food Consumption Rate Questionnaire among Consumers (FCRQ); Table S2: Conceptual appraisal and item refinement decisions for the 20 FCRQ items; Table S3: Summary of conceptual review and item refinement decisions for the 20 FCRQ items; Table S4: Average Variance Extracted (AVE) and Composite Reliability (CR); Table S5: Heterotrait–Monotrait Ratio (HTMT); Table S6: Fornell–Larcker Criterion; Table S7: Summary Table (Loadings Range, AVE, CR, HTMT); Table S8: Convergent Validity Indices (AVE & CR) for the Four FCRQ Factors; Table S9: Discriminant Validity of the FCRQ (Fornell–Larcker Criterion & HTMT Ratios); Table S10: Sociodemographic characteristics of the study sample (N = 555); Table S11: Mean Values and Standard Deviations for All Items Assessing Food Consumption; Table S12: Gender-stratified item-level descriptive statistics (Mean ± SD) for all 20 FCRQ items; Table S13: Pattern Matrix of the FCRQ (Principal Axis Factoring, Promax Rotation).
