# Supplementary material for: Development and Validation of the Food Consumption Rate Questionnaire (FCRQ): A Behaviorally Informed Measure of Eating-Rate Regulation
Source: Nutrients. 2026 Aug 12;18(16):2637. doi: 10.3390/nu18162637 (PMC13516422; doi:10.3390/nu18162637)
Supplement: Supplementary file 1 [file nutrients-18-02637-s001.zip › nutrients-4491768-supplementary.pdf]

Development and Validation of the Food Consumption Rate Questionnaire (FCRQ): A Behaviorally Informed Measure of Eating-Rate Regulation

Maria P. Koliou [1], Chrysoula Karaiskou [2], Charalampos Eleftheriadis [2] and Dimitris Skalkos [1],*

[1]Laboratory of Food Chemistry, Department of Chemistry, University of Ioannina, 45110 Ioannina, Greece; m.koliou@uoi.gr

[2] Sidroco Holdings Ltd., Nicosia 1082, Cyprus; ckaraiskou@sidroco.com (C.K.); celeftheriadis@sidroco.com (C.E.)

* Correspondence: dskalkos@uoi.gr; Tel.: +30-2651008345

Supplementary material

The Supplementary Materials include the full questionnaire and detailed psychometric tables supporting the analyses presented in the manuscript.

Figure S1. Conceptual model illustrates the emotional, sensory, regulatory, and contextual factors influencing eating rate, along with their downstream effects on behavioral outcomes, dietary choices, weight status, and overall health impacts.

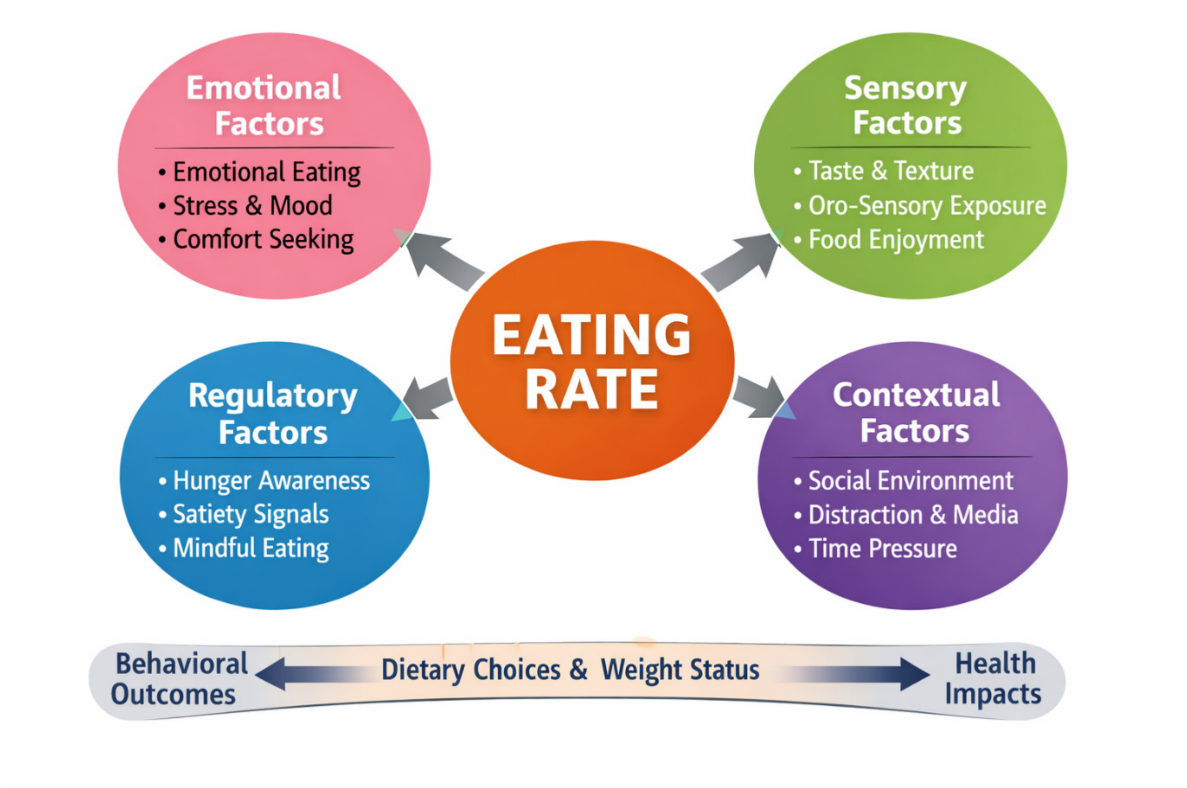

Table S1

Questionnaire on Food Consumption Rate Questionnaire among Consumers (FCRQ)

QUESTIONNAIRE

FOOD CONSUMPTION RATE

I. Demographic Information

1. Gender
Male
Female

2. Age
16-18
19-22
23-28

3. Employment / Study Status
Working student
Full-time student

4. Region of permanent residence in Greece
Northern Greece (Macedonia – Thrace)
Western Greece (Epirus – Aetolia-Acarnania)
Central Greece (including Athens)
Peloponnese
Islands (Aegean and Ionian)

Please indicate your level of agreement with each statement using a 5-point Likert scale ranging from **1 = Strongly Disagree** to **5 = Strongly Agree**. A score of **1** represents **strong disagreement** with the statement, while a score of **5** represents **complete agreement**. A score of **3** indicates a **neutral response**, due to uncertainty or ambivalence.

(Please mark your answer with an "X".)

1. Slowness in Food Consumption

Compared to usual	Strongly Disagree	Disagree	Neutral	Agree	Strongly Agree
1. I eat slowly	☐ 1	☐ 2	☐ 3	☐ 4	☐ 5
2. I am usually the last person to finish my meal	☐ 1	☐ 2	☐ 3	☐ 4	☐ 5
3. I often finish my meals very quickly	☐ 1	☐ 2	☐ 3	☐ 4	☐ 5
4. During a meal, I tend to eat increasingly slowly	☐ 1	☐ 2	☐ 3	☐ 4	☐ 5
5. My eating pace is generally slow compared to others	☐ 1	☐ 2	☐ 3	☐ 4	☐ 5

2. Awareness of Eating Rate

Compared to usual	Strongly Disagree	Disagree	Neutral	Agree	Strongly Agree
1. I can easily perceive how fast or slow I am eating	☐ 1	☐ 2	☐ 3	☐ 4	☐ 5
2. I notice when my eating rate changes	☐ 1	☐ 2	☐ 3	☐ 4	☐ 5
3. I know when I am eating faster than usual	☐ 1	☐ 2	☐ 3	☐ 4	☐ 5
4. I am able to control the pace at which I eat	☐ 1	☐ 2	☐ 3	☐ 4	☐ 5
5. The way I eat is influenced by how consciously I monitor my pace	☐ 1	☐ 2	☐ 3	☐ 4	☐ 5

3. External Factors and Eating Rate

Compared to usual	Strongly Disagree	Disagree	Neutral	Agree	Strongly Agree
1. I eat faster when I am with company	☐ 1	☐ 2	☐ 3	☐ 4	☐ 5
2. I eat more slowly when I am in a calm environment	☐ 1	☐ 2	☐ 3	☐ 4	☐ 5
3. The presence of other people affects the pace at which I eat	☐ 1	☐ 2	☐ 3	☐ 4	☐ 5
4. When I am in a hurry, I tend to eat very quickly	☐ 1	☐ 2	☐ 3	☐ 4	☐ 5
5. Noise or surrounding intensity affects how fast I eat	☐ 1	☐ 2	☐ 3	☐ 4	☐ 5

4. Awareness and Enjoyment of Eating

Compared to usual	Strongly Disagree	Disagree	Neutral	Agree	Strongly Agree
1. When I eat slowly, I enjoy my food more	☐ 1	☐ 2	☐ 3	☐ 4	☐ 5
2. Eating slowly helps me better recognize the feeling of fullness	☐ 1	☐ 2	☐ 3	☐ 4	☐ 5
3. When I eat quickly, I find it difficult to understand when I am full	☐ 1	☐ 2	☐ 3	☐ 4	☐ 5
4. Conscious, slow eating makes me feel more satisfied after a meal	☐ 1	☐ 2	☐ 3	☐ 4	☐ 5
5. Slow eating contributes to healthier food choices	☐ 1	☐ 2	☐ 3	☐ 4	☐ 5

Table S2. Conceptual appraisal and item refinement decisions for the 20 FCRQ items.

Item Code	Decision	Notes
Q1_1	Retained	Clear wording; aligns with slowness construct
Q1_2	Refined	Minor rewording for clarity
Q1_3*	Retained	Reverse-coded item capturing fast eating; conceptually essential
Q1_4	Refined	Minor refinement to improve precision
Q1_5	Retained	Strong conceptual alignment
Q2_1	Retained	Good theoretical fit with awareness construct
Q2_2	Retained	Clear and concise
Q2_3	Retained	Slight conceptual overlap with Q2_5 but distinct enough
Q2_4	Retained	Strong conceptual relevance
Q2_5	Retained	Maintains conceptual breadth of awareness domain
Q3_1	Retained	Clear representation of external factors
Q3_2	Retained	Good contextual relevance
Q3_3	Retained	Strong alignment with environmental modulation
Q3_4	Retained	Captures social facilitation influences
Q3_5	Retained	Clear and theoretically grounded
Q4_1	Retained	Good representation of mindful engagement
Q4_2	Retained	Strong conceptual clarity
Q4_3	Retained	Captures sensory enjoyment
Q4_4	Retained	Good alignment with fullness recognition
Q4_5	Retained	Slightly broader wording but conceptually important

* Reverse-coded item. Scoring direction was inverted to ensure consistency across the Slowness in Food Consumption domain

Table S3. Summary of conceptual review and item refinement decisions for the 20 FCRQ items

Item Code	Conceptual Comment	Modification Applied
Q1_1	Wording slightly ambiguous; comparative phrasing could be clearer	Reworded for clarity
Q1_2	Conceptually clear; no overlap with other items	No modification needed
Q1_3*	Important behavioral indicator capturing fast eating; scoring direction required reversal	Reverse-coded; retained due to conceptual importance
Q1_4	Clear item but could benefit from more specific phrasing	Minor refinement applied
Q1_5	Strong conceptual fit; captures perceived slowness effectively	No modification needed
Q2_1	Strong theoretical alignment; essential for awareness domain	No modification needed
Q2_2	Clear and concise; captures interoceptive awareness effectively	No modification needed
Q2_3	Partial conceptual overlap with Q2_5	Minor rephrasing to reduce redundancy
Q2_4	Excellent clarity; strong conceptual relevance	No modification needed
Q2_5	Slightly broad wording; important for conceptual breadth	Wording improved; retained
Q3_1	Represents environmental influence well	No modification needed
Q3_2	Clear but context could be specified more explicitly	Added contextual descriptor
Q3_3	Good fit; captures social facilitation	No modification needed
Q3_4	Environmental cue not clearly defined	Wording refined for specificity
Q3_5	Strong item; captures external modulation effectively	No modification needed
Q4_1	Clear and theoretically aligned	No modification needed
Q4_2	Strong conceptual clarity; central to enjoyment domain	No modification needed
Q4_3	Captures mindful engagement; no issues	No modification needed
Q4_4	Slightly broad wording; could be interpreted variably	Minor refinement applied
Q4_5	Broad interpretation possible; conceptually important	Wording narrowed; retained

* Reverse-coded item. Scoring direction was inverted to ensure consistency across the Slowness in Food Consumption domain

Table S4. Average Variance Extracted (AVE) and Composite Reliability (CR)

Factor	AVE	CR
Slowness	0.67	0.91
Awareness of Eating Rate	0.30	0.64
External Factors	0.27	0.62
Awareness & Enjoyment	0.45	0.80

Table S5. Heterotrait–Monotrait Ratio (HTMT)

Factor Pair	HTMT
Slowness – Awareness	0.41
Slowness – External	0.38
Slowness – Enjoyment	0.52
Awareness – External	0.36
Awareness – Enjoyment	0.48
External – Enjoyment	0.44

Table S6. Fornell–Larcker Criterion

Factor	√AVE	Slowness	Awareness	External	Enjoyment
Slowness	0.82	0.82	0.15	0.18	0.32
Awareness	0.55	0.15	0.55	0.15	0.22
External Factors	0.52	0.18	0.15	0.52	0.22
Awareness & Enjoyment	0.67	0.32	0.22	0.22	0.67

Table S7. Summary Table (Loadings Range, AVE, CR, HTMT)

Factor	Items	Loadings Range	AVE	CR	HTMT (max)
Slowness	5	0.515–0.911	0.67	0.91	0.52
Awareness	5	0.145–0.705	0.30	0.64	0.48
External Factors	5	0.190–0.668	0.27	0.62	0.44
Awareness & Enjoyment	5	0.488–0.824	0.45	0.80	0.52

Table S8. Convergent Validity Indices (AVE & CR) for the Four FCRQ Factors. [*Convergent validity was assessed using Average Variance Extracted (AVE) and Composite Reliability (CR). Values were calculated using standardized factor loadings from the CFA model].*

Factor	Items	AVE	CR
Slowness in Food Consumption	Q1_1–Q1_5*	0.57	0.89
Awareness and Enjoyment of Eating	Q4_1–Q4_5	0.49	0.82
Awareness of Eating Rate	Q2_1–Q2_5	0.41	0.74
External Factors and Eating Rate	Q3_1–Q3_5	0.29	0.63
* Q1_3 is reverse-coded to ensure consistent scoring direction within the factor.			

Table S9. Discriminant Validity of the FCRQ (Fornell–Larcker Criterion & HTMT Ratios). *[Discriminant validity was evaluated using the Fornell–Larcker criterion and the Heterotrait–Monotrait (HTMT) ratio. All √AVE values exceeded inter-factor correlations, and all HTMT values were below the recommended threshold of 0.85, indicating adequate discriminant validity].*

Fornell–Larcker Matrix

Factor	√AVE	F1	F2	F3	F4
F1. Slowness in Food Consumption	0.75	0.75	0.384	0.221	0.087
F2. Awareness of Eating Rate	0.70	0.384	0.70	0.310	0.145
F3. External Factors and Eating Rate	0.64	0.221	0.310	0.64	0.176
F4. Awareness and Enjoyment of Eating	0.54	0.087	0.145	0.176	0.54

HTMT Ratios

Factor Pair	HTMT
F1–F2	0.18
F1–F3	0.17
F1–F4	0.27
F2–F3	0.19
F2–F4	0.18
F3–F4	0.34

Table S10. Sociodemographic characteristics of the study sample (N = 555).

Characteristic	n	%
**Gender**		
Male	130	23.4
Female	423	76.2
Other / Not specified	2	0.4
**Age group**		
16–18	2	0.4
19–22	375	67.6
23–28	127	22.9
**Professional status**		
Working student	122	22.0
Full-time student	432	77.8
Other / Not specified	1	0.2
**Region of permanent residence**		
Northern Greece	146	26.3
Western Greece (Epirus–Aitoloakarnania)	186	33.5
Central Greece (incl. Athens)	143	25.8
Islands (Aegean & Ionian)	40	7.2
Peloponnese	38	6.8

Missing Data Considerations. The total sample consisted of 555 participants. For certain variables (age group and region of permanent residence), not all participants provided a response; therefore, n and percentages are based on valid responses only.

Table S11. Mean Values and Standard Deviations for All Items Assessing Food Consumption. *[Values range from 1 (strongly disagree) to 5 (strongly agree). Response counts vary slightly across items (N = 552–554)].*

Question Code	Item Description	Mean Value	Standard Deviation
Q1_1	I eat slowly	2.95	1.13
Q1_2	I am usually the last person to finish my meal	2.79	1.27
Q1_3*	I often finish my meals very quickly	2.95	1.23
Q1_4	During a meal, I tend to eat increasingly slowly	2.70	0.98
Q1_5	My eating pace is generally slow compared to others	2.78	1.17
Q2_1	I can easily perceive how fast or slow I am eating	3.64	0.93
Q2_2	I notice when my eating rate changes	3.59	0.89
Q2_3	I know when I am eating faster than usual	3.95	0.81
Q2_4	I am able to control the pace at which I eat	3.30	0.96
Q2_5	The way I eat is influenced by how consciously I monitor my pace	3.09	1.01
Q3_1	I eat faster when I am with company	2.39	0.94
Q3_2	I eat more slowly when I am in a calm environment	3.35	1.04
Q3_3	The presence of other people affects the pace at which I eat	3.56	1.03
Q3_4	When I am in a hurry, I tend to eat very quickly	4.18	0.87
Q3_5	Noise or surrounding intensity affects how fast I eat	2.97	1.07
Q4_1	When I eat slowly, I enjoy my food more	3.50	1.02
Q4_2	Eating slowly helps me better recognize the feeling of fullness	3.72	0.94
Q4_3	When I eat quickly, I find it difficult to understand when I am full	3.55	1.06
Q4_4	Conscious, slow eating makes me feel more satisfied after a meal	3.46	1.00
Q4_5	Slow eating contributes to healthier food choices	3.51	1.07
* Reverse-coded item. Scoring direction was inverted to ensure consistency within the Slowness in Food Consumption factor			

Table S12. Gender-stratified item-level descriptive statistics (Mean ± SD) for all 20 FCRQ items. *[Values reflect agreement on a 5-point Likert scale (1 = strongly disagree, 5 = strongly agree)].*

Item	Male (M ± SD)	Female (M ± SD)
Q1_1	2.65 ± 1.13	3.04 ± 1.12
Q1_2	2.32 ± 1.16	2.95 ± 1.26
Q1_3*	3.46 ± 1.09	2.79 ± 1.22
Q1_4	2.66 ± 0.97	2.71 ± 0.99
Q1_5	2.41 ± 1.10	2.90 ± 1.17
Q2_1	3.72 ± 0.96	3.62 ± 0.92
Q2_2	3.58 ± 0.90	3.60 ± 0.89
Q2_3	3.88 ± 0.85	3.97 ± 0.80
Q2_4	3.45 ± 0.97	3.26 ± 0.95
Q2_5	3.06 ± 1.03	3.10 ± 1.00
Q3_1	2.41 ± 0.92	2.39 ± 0.95
Q3_2	3.25 ± 1.11	3.38 ± 1.02
Q3_3	3.48 ± 1.06	3.59 ± 1.02
Q3_4	4.29 ± 0.79	4.14 ± 0.90
Q3_5	2.86 ± 1.01	3.00 ± 1.08
Q4_1	3.22 ± 1.12	3.58 ± 0.98
Q4_2	3.45 ± 0.97	3.80 ± 0.92
Q4_3	3.25 ± 1.08	3.64 ± 1.04
Q4_4	3.12 ± 1.10	3.57 ± 0.95
Q4_5	3.34 ± 1.05	3.57 ± 1.07

* Reverse-coded item. Scoring direction was inverted to ensure consistency within the Slowness in Food Consumption factor.

Table S13. Pattern Matrix of the FCRQ (Principal Axis Factoring, Promax Rotation). *[Factor loadings < 0.30 are suppressed for clarity].*

Item	Factor 1	Factor 2	Factor 3	Factor 4
Q1_5	0.914			
Q1_2	0.884			
Q1_3*	0.854			
Q1_1	0.842			
Q1_4	0.469			
Q4_4		0.827		
Q4_2		0.733		
Q4_3		0.637		
Q4_1		0.611		
Q4_5		0.465		
Q2_3			0.693	
Q2_1			0.692	
Q2_2			0.605	
Q2_4			0.372	
Q3_5				0.667
Q3_3				0.469
Q3_4				0.395
Q3_2	0.362			0.314

* Reverse-coded item. Scoring direction was inverted to ensure consistency within the Slowness in Food Consumption factor
